# COVID-19 and Organ Transplantation

**Published:** 2020

**Authors:** B. Joob, V. Wiwanitkit

**Affiliations:** 1 *Sanitation1 Medical Academic Center, Bangkok, Thailand*; 2 *Honorary Professor, dr DY Patil University, Pune, India, and Visiting Professor, Hainan Medical University, Haikou, China*

**DEAR EDITOR,**

COVID-19 is an emerging viral infection. It originated from China; it then progressed to Thailand and other countries including Iran [1, 2]. Thousands of cases have been reported worldwide. There are many patients with underlying medical risks. Herein, we would like to focus on those factors relevant to transplantation medicine. So far, there is no official report of infection in patients who underwent organ transplantation. In MERS, another emerging coronavirus infection, organ transplant recipients could be infected, but rarely [3].

The infection is still in its first few months, and it mainly affects travelers. Therefore, the chance for organ transplant recipients is very low. However, in a pandemic, the infection might also affect organ transplant recipients. Another hypothesis would be the potential protective effect of immunosuppressive drugs and prophylactic chemotherapy used by organ transplant recipients against the disease.

## CONFLICTS OF INTEREST:

**None declared.**
